# Rights vs. Lived Realities: Women’s Views of Gender Equality in Relationships in Rural South Africa

**DOI:** 10.1093/socpro/spac015

**Published:** 2022-03-03

**Authors:** Christie Sennott, Danielle Kane

**Affiliations:** Purdue University

**Keywords:** gender inequality, Africa, rights, HIV, violence

## Abstract

South Africa’s Constitution is among the world’s most ambitious in promoting gender equality, but the country continues to be marked by inequality and gender-based violence. Given this context, we analyze 43 interviews with Black women aged 18-55 in rural South Africa to explore how the constitutional ideal of gender equality—or “50/50”—has been interpreted and applied in women’s intimate relationships. Overall, we found that inequality and gender hierarchy were common in relationships. Women relied on two logics to explain the persistence of inequality in their relationships. First, women offered ideological support for gender norms supporting hierarchy by linking 50/50 to the abandonment of culture, tradition, and respect. Second, women viewed reaffirmation of gender inequality within relationships as a pragmatic way to avoid men’s violence and infidelity, thus protecting women from abandonment and HIV. Women’s views about equality in relationships were shaped by dominant gender norms, precarity in the local political economy, and the risks of violence and HIV/AIDS. Our findings expand theories of social change by highlighting how not only longstanding social norms, but also local political-economic and health conditions can influence views of equality and ultimately the local adoption or dismissal of international standards of rights and equality.

Gender equality and the empowerment of all women and girls is the fifth United Nations Sustainable Development Goal, which countries around the world are working toward meeting by 2030 (United Nations 2020). Despite progress over the past several decades, gender inequality in both the public and private spheres remains a significant social problem across the globe (World Bank 2020). Thus, scholars and policymakers have called for more attention to gender equality as a public good, for reducing health disparities, and as a key driver of economic development (see for example Baten and de Plejit 2019; Helman and Ratele 2016; Kabeer 2016).

The current study examines women’s views of gender equality in intimate relationships in South Africa, a country in which progressive democratic ideals coexist with extreme inequality (World Bank 2019). The end of the apartheid system of government in 1994 witnessed the adoption of a new South African Constitution, stipulating not only racial equality but also a host of other progressive social policies, including gender equality (see Hassim 2006, 2018). While many new democracies have taken only nominal steps toward increasing gender equality, South Africa aimed to create “a truly inclusive polity…[that] involved a remarkable effort to confront gender inequalities” (Seidman 2001:220). Given the governmental support for equal rights, there was an assumption that “real change in a feminist direction was possible through the state” (Hassim 2003:505). Indeed, explicit governmental support for gender equality has continued, evidenced most recently by the 2020 passage of the Emergency Response Action Plan and the introduction of several new bills to combat gender-based violence and femicide.^1^

Despite the institutional support for gender equality, however, there remains a vast divide between “South Africa’s formal status as a ‘rights paradise’ and the grim realities of people’s daily lives” (Robins 2008:2). Following the transition to democracy and the widespread promotion of women’s rights, researchers documented a “crisis in masculinity” (Walker 2005:226) and backlash against women, contributing to high rates of intimate partner violence (IPV) and HIV (Jewkes et al. 2010; Seedat et al. 2009). Though significant progress has been made in HIV testing and treatment programs, South Africa continues to have the world’s largest HIV/AIDS epidemic (UNAIDS 2018) with a much higher prevalence among women (26 percent) than men (15 percent) (HSRC 2018). Additionally, estimates suggest nearly one-third (30 percent) of women in South Africa have experienced recent IPV (UNAIDS 2018). These social and health epidemics have prompted numerous interventions aimed at curbing gender-based violence and reducing women’s risk of HIV, in part by altering gender norms that position women as subordinate to men (Dworkin et al. 2012; Gibbs et al. 2015, 2020; Jewkes and Morrell 2010; Willan et al. 2020).

In this study, we explore the disconnect between South Africa’s governmental commitment to equal rights and women’s views of gender equality in intimate relationships, referred to colloquially as “50/50”. To do so, we analyze 43 in-depth interviews with Black women in rural Mpumalanga Province, South Africa. Our research is grounded in work that addresses how international discourses of rights get (re)interpreted at the local level (e.g., Kurzman et al. 2019; Thomas 2007; Unnithan and Pigg 2014) by examining how women interpret and apply ideals of equal rights in their relationships. This approach also aligns with evidence from sub-Saharan Africa that the backlash to women’s rights often plays out within relationships (e.g., Jewkes et al. 2010; Pierotti, Lake, and Lewis 2018; Walker 2005; Wyrod 2016). Overall, we found that inequality and gender hierarchy were common in intimate relationships. Women relied on two logics to explain the persistence of inequality in their relationships. First, women offered ideological support for longstanding norms supporting gender hierarchy by linking 50/50 to the abandonment of culture, tradition, and respect. Second, women viewed the reaffirmation of gender inequality in relationships as a pragmatic way to avoid men’s violence and infidelity, thus protecting women from abandonment and HIV. Taken together, women’s views about equality in relationships were shaped by dominant gender norms, precarity in the local political economy, and the significant risks of violence and HIV. Our findings highlight the importance of social and cultural factors as well as the material conditions that structure life in rural areas in how ideals of equal rights are adopted or dismissed in relationships.

This study is one of the first in rural sub-Saharan Africa to ask women directly about their views of gender equality in relationships. Although research from the United States has documented attitudes among men and women signaling a stalled gender revolution (e.g., England, Levine, and Mishel 2020), there are few comparable studies of women in sub-Saharan Africa, where equal rights were established more recently and societies continue to undergo rapid social change. Additionally, although there is a growing body of research examining men’s responses to women’s rights in sub-Saharan Africa (e.g., Dworkin et al. 2012; Dworkin, Fleming, and Colvin 2015; Pierotti et al. 2018; Wyrod 2008), few studies have analyzed the factors influencing women’s own views of gender equality in relationships (see Pettifor et al. 2012; Wyrod 2016 for exceptions). The current study contributes to this work by focusing on a distinct rural setting marked by structural disadvantage in which narratives about equal rights and a reassertion of tradition both compete and intertwine (Ainslie and Kepe 2016; Mathis 2011), allowing us to parse out the local dynamics that are often obscured in national-level survey data. Additionally, our in-depth interviews provide insight into the processes through which ideals of equality are interpreted and applied within the context of women’s relationships. This approach is valuable for better understanding the reasons why legislating gender equality does not always translate into changes in women’s lived realities.

## Background

Explanations for the persistence of gender inequality often hinge on longstanding social and cultural norms that position women as subordinate to men (see for example Ridgeway 2011). More broadly, norms scholars argue that because social norms—or group-level expectations for behavior and the sanctions for breaking them—are fundamental to societies, they have a way of persisting and maintaining inequality even in the face of significant social change (Horne and Mollborn 2020). Individual beliefs and behaviors are influenced by norms, which become internalized (Horne and Mollborn 2020; Risman 2018). Once internalized, these expectations for behavior become a routine and taken for granted part of how individuals evaluate their own actions and the actions of others, similar to what Risman calls “the cultural component of the social structure” or “the interactional expectations that each of us meet in every social encounter” (2004:433; see also Giddens 1984; Horne and Mollborn 2020). Although structural changes promoting gender equality might be expected to alter gender hierarchy and interactions between men and women, Ridgeway argues individuals bring “trailing gender beliefs” into new social contexts, reinscribing the status assumptions tied to these beliefs onto new social arrangements and ultimately reinforcing existing inequalities (2011:28). This suggests that analyses centering on how individuals interpret and apply abstract ideals of gender equality into the fabric of their everyday lives may be particularly fruitful for understanding the persistence of inequality in the face of social change (Risman 2004), such as the documented disconnect between policy change in South Africa and resistance on the ground. This also allows us to see how women employ agency in making decisions to maximize their security and wellbeing within restrictive cultural and social norms (Kandiyoti 1988; Mathis 2011; Mphaphuli and Smuts 2021; Willan et al. 2020), rather than viewing women simply as victims of inequality.

### Ideological Support for Inequality

Normative systems of hegemonic masculinity and emphasized femininity reinforce gender inequality by dictating complementary and hierarchical relations between men and women (Connell 1987, 1995). More specifically, although they are not monolithic, hegemonic constructions of masculinity across South Africa emphasize dominance, control, sexual prowess, and economic provision (Hunter 2005; Jewkes and Morrell 2012), whereas dominant constructions of emphasized or acquiescent femininity emphasize deference, modesty, respectability, and caretaking (Bhana 2016; Gqola 2007; Harrison 2008; Jewkes and Morrell 2012; Sennott and Mojola 2017). Because these norms reinforce gender hierarchy and inequality, women’s fulfilment of the social and cultural expectations surrounding femininity effectively subordinates them to men (Bhana 2016; Harrison 2008; Jewkes and Morrell 2012; Mathis 2011; Mphaphuli and Smuts 2021; Sennott and Mojola 2017). The persistence of dominant gender norms has contributed to high rates of IPV and HIV, prompting several interventions across South Africa. For example, the One Man Can Programme, implemented by the South African NGO Sonke Gender Justice, and the Stepping Stones, Creating Futures Programme, implemented by the South African Medical Research Council and Project Empower, focus on altering norms, beliefs, and behaviors related to masculinity (see van den Berg et al. 2013; Dworkin et al. 2012; Gibbs et al. 2015, 2020; Ratele 2015; Treves-Kagan et al. 2020; Willan et al. 2020). Nonetheless, research has documented resistance among men to ideals of equality due to the assumed tradeoff between improvements in women’s status and a loss of men’s power and respect (Dworkin et al. 2012; Posel 2004; Walker 2005). Thus, equality is viewed through a zero-sum approach in which women’s gains equal men’s losses. For example, research from South Africa found that men viewed women’s empowerment and increasing access to jobs and money as disruptive to gender relations and disempowering for men (Dworkin et al. 2012). Similar dynamics have been noted in other sub-Saharan African settings such as Uganda where men reported feeling victimized and disempowered due to efforts to promote women’s rights and equality (Wyrod 2008, 2016) and in the Democratic Republic of Congo where men who participated in gender sensitization workshops were somewhat more willing to share domestic tasks, but not power (Pierotti et al. 2018). Taken together, these studies highlight how men’s compliance with hegemonic masculinity can impede the acceptance of women’s rights and gender equality.

### Tensions in Women’s Support for Equality

Research has shown that dominant gender norms can also influence women’s beliefs and actions regarding equality in relationships. Specifically, women may implicitly support hegemonic masculinity through their enactments of femininity (Jewkes and Morrell 2012; Schippers 2007). For example, women who idealize men who fulfill the provider role and therefore encourage men to enact this behavior buttress the power of norms that reinforce women’s dependence on men (Bhana and Patttman 2011; Pettifor et al. 2012; Talbot and Quayle 2010). Though “modern” or “resistant” femininities that reject gender hierarchy and women’s submission are becoming more prevalent across South Africa, women who push for equality are typically viewed as challenging dominant norms, thereby incurring rebuke from families, friends, and communities (Dworkin et al. 2015; Jewkes and Morrell 2012; Pettifor et al. 2012; Sennott and Angotti 2016). This may encourage women to temper their support for equality in relationships or dismiss changes that would threaten what is often perceived as the “natural” gender order. For example, although women in urban Uganda were critical of men’s abuses of power, they remained emphatic that men should have more power and authority because these traits were given to men “by God” (Wyrod 2008). Further, women may be compelled to abide by norms of acquiescent femininity if there are few other options or if doing so provides women with respect, status, and security (Graham 2016; Jewkes and Morrell 2012; Kandiyoti 1988; Sennott and Mojola 2017). These dynamics put pressure on women to adhere to dominant gender norms dictating their own submission (see also Campbell and Nair 2014; Ranganathan et al. 2021).

We build on this work by focusing specifically on how women view and navigate ideals of equal rights within their relationships. In doing so, we aim to identify local factors that create tension in women’s support for gender equality. This is critical as scholars have argued that universal notions of rights—such as those adopted by the South African government—lack grounding in women’s lived experiences; therefore, accounting for local factors and structures is necessary to understand why legislated rights may not translate into equality on the ground (see Undie and Izugbara 2011). Further, research from South Africa has highlighted the importance of recognizing “the everyday complexities of women’s lives, including the patriarchal social structures that constrain them” (Ranganathan et al. 2021:3). Thus, despite the government’s commitment to gender equality across all realms of society—including the family—local cultural frameworks may still encourage the belief that men deserve more rights and power than women (Thomas 2007).

In rural South Africa, high levels of economic precarity (Blalock 2014; Statistics South Africa 2020) and the ubiquity of HIV/AIDS (Gómez-Olivé et al. 2013) are important local conditions likely to affect how women weigh equality in relationships vis-à-vis their own security and wellbeing. Additionally, because of its prevalence, IPV constitutes a significant public health concern and is closely tied to men’s fulfilment of hegemonic masculinity norms and the risk of HIV (Dunkle et al. 2004; Jewkes et al. 2010; Treves-Kagan et al. 2020). Research from South Africa has shown that defying dominant norms can be detrimental for women’s security, respectability, health, and wellbeing in the context of economic precarity and uncertainty due to HIV/AIDS (Ratele 2015; Sennott, Madhavan, and Nam 2020). Building on this work, we examine women’s views about gender equality in relationships as a window into the local norms, political-economic structures, and health uncertainties that shape why women’s rights and equality are adopted or dismissed.

## Methods

This study is nested in the Agincourt Health and Socio-Demographic Surveillance System (Agincourt) site in rural Mpumalanga Province, South Africa. Agincourt incorporates approximately 110,000 individuals in 21,000 households in 31 villages and has conducted an annual census of demographic and health information since 1992 (see www.agincourt.co.za). The majority of Agincourt residents are of the Shangaan ethnic group and one-third are of Mozambican origin. The area is a former apartheid-era “homeland” where Black South Africans were forced to resettle and endured the compounding hardships of inadequate healthcare, employment, education, and infrastructure (Worden 2007). The area still has limited resources: water is provided through neighborhood taps, there is no formal sanitation system, and electricity is unreliable and expensive (Kahn et al. 2012). Challenges in the local political economy have rendered most paid work temporary, informal, and underpaid, leading to high unemployment (Blalock 2014; Statistics South Africa 2020). These economic challenges push many men and increasingly women to out-migrate for work (Blalock 2014). HIV/AIDS is endemic in the area: prevalence is nearly 20 percent among adults, with a much higher rate among women (24 percent) than men (11 percent) (Gómez-Olivé et al. 2013). Our findings reflect how these local conditions influence rural women’s beliefs about gender equality.

The larger study from which this analysis is drawn was designed to explore rural South African women’s views and experiences related to relationship formation, marriage processes, gender equality in relationships, and health and wellbeing (see also Sennott et al. 2020). In 2015, a research team comprised of the first author and three local research assistants conducted in-depth interviews in XiShangaan/XiTonga (local language) with 43 Black women aged 18-55 in Agincourt. All participants provided written informed consent. The interview protocol was semistructured, allowing for probing on issues unique to participants. Interviews lasted 1-2 hours, were audio-recorded, and translated and transcribed by the research team. The findings drawn from the interview data were supported by the first author’s extensive experiences working in this area of South Africa. The study received ethical approval from the Mpumalanga Province Department of Health, local leadership, and university institutional review boards in the United States and South Africa. We use pseudonyms to protect the identities of participants.

Participants were recruited through a quota snowball sampling technique based on recommendations from a Community Advisory Group that advises on best practices in the site, local research assistants’ social networks, and participants’ referrals of the study to other women. Because marriage is an important marker of women’s status and wellbeing in Agincourt, the aim was to recruit roughly equal numbers of women based on relationship status and bridewealth (“lobola” in Shangaan). The analytic sample included 13 women (30 percent of the sample) who were married with lobola paid, 13 women (30 percent) who were cohabiting with a partner without lobola, and 17 women (40 percent) who were neither married nor cohabiting (labeled “single” below). Participants were 34 years old on average. Nearly 70 percent had finished secondary school and 26 percent were employed in either the formal or informal labor market. Additionally, one woman was volunteering at a preschool, one was in the process of opening a small business, and one was in school. One woman was pregnant and participants had 2.6 children on average (range 0-9), consistent with fertility patterns in Agincourt (Williams et al. 2013).

We used a combination of deductive and inductive coding strategies to analyze the data (Charmaz 2001; Strauss and Corbin 1990). We first engaged in structured coding by interview question, focusing primarily on the question: “Do you think men and women should be equal in their relationships? Why/why not?” We also analyzed several follow-up questions addressing the good and bad things about 50/50; whether women’s own relationships were 50/50; and significant others’ views of 50/50. Thus, interviews focused on how ideals about gender equality were interpreted and applied in intimate relationships and did not engage in broader discussions about how rights and equality function across other societal spheres (see Hames 2006 for a discussion of the latter). Although the term “50/50” was introduced by the South African government and is often used publicly to refer to gender representation in government and employment, it has been appropriated at the local level to refer to equality in relationships and households, as several studies have reported (e.g., Dworkin et al. 2012; Hunter 2010; Pettifor et al. 2012).

After structured coding, we engaged in inductive coding based on emergent themes and found an overarching pattern of women reaffirming gender hierarchy and inequality in relationships. Based on this theme, we returned to the data to reanalyze and recode the transcripts. Through this iterative process, we found no systematic differences in views of gender equality based on women’s socio-demographic characteristics. The quotes provided below illustrate the most prevalent themes across interviews. To aid in readability of the quotes, we made minor edits to grammar and include clarifying phrases in brackets.

## Findings

We found that inequality and gender hierarchy were common in intimate relationships. Women relied on two logics to explain the persistence of gender inequality in their relationships. First, 86 percent (n=37) of the sample offered ideological support for longstanding norms supporting gender hierarchy by linking 50/50 to the abandonment of culture, tradition, and respect. This explanation drew on gender essentialist scripts defining differences between men and women and men as “natural” and unmalleable. Second, 77 percent (n=33) viewed the reaffirmation of gender hierarchy in relationships as a pragmatic way to avoid men’s violence and infidelity, thus protecting women from abandonment and HIV. This explanation highlighted local political-economic uncertainties and health risks and emphasized that 50/50 relationships were available only to women who were highly educated and employed, and thus not economically dependent on men. Overall, 67 percent (n=29) provided both ideological and pragmatic explanations for reaffirming gender inequality in relationships and 95 percent (n=41) relied on at least one of these logics. Four participants (9 percent) were ideologically supportive of gender *equality* in relationships but did not fully support it because of pragmatic barriers. Only two participants^2^ offered full support of gender equality in relationships. Taken together, women’s views about equality in relationships were shaped by dominant gender norms, precarity in the local political economy, and the significant risks of violence and HIV.

### Ideological Explanations for Gender Inequality

Women often explained their reaffirmation of gender inequality in relationships by relying on scripts essentializing differences between men and women, reinforcing gender hierarchy, and linking women’s fulfilment of dominant gender norms to respect, culture, and tradition. When asked whether men and women should be equal in relationships, Alice (age 37, married) said: “No, they are not equal. A man is the head of the family. Even when a man and a woman are both working, a man will always be a man.” Similarly, Alina (age 44, married) supported gender hierarchy by emphasizing her partner’s position as household head and women’s duty to comply with the gender order:

We are not equal, and he [her partner] also has more power than me because I am a woman. So, a woman must always humble herself to her man. A man will always be a man and it will never change. Even if a woman can have more money, it will never change. A man will always be head of the family.

Alice’s and Alina’s comments emphasize ideological support for gender hierarchy over pragmatic concerns in arguing that even women who are employed—and may have some measure of economic independence—must “humble” themselves or be dutiful to their partners. This belief reinforces men’s “natural” position over women in relationships, as reflected in Alina’s claim that her husband has more “power” than her as a woman. Further, each woman’s quote followed a gender essentialist script by reinforcing the permanence of gender difference (“always”, “never”). As Tengisa (age 19, single) succinctly summed up: “It’s [50/50] bad because a man will always be a man, and [a] woman will always be a woman. They will never be equal.”

The ideological support women offered for gender hierarchy and inequality in relationships was supported by dominant gender norms, which were held sacred among community members and buttressed by local institutions, including churches. For example, women frequently invoked the Bible or their pastor’s teachings in explaining their views of 50/50 in relationships. As Nyeleti (age 26, cohabiting) put it:

Nyeleti: Our pastor is saying we must not live by 50/50. A man should always remain a man. He is the head of the family. He [the pastor] is saying in the beginning God created a man and the second one was a woman. Women are after men in everything.

Interviewer: How do women feel about this preaching?

Nyeleti: We do understand and accept that. We also pray to God to help us live according to the pastor’s preachings.

By teaching that gender essentialism and hierarchy are ordained by God, some pastors participate in perpetuating “an inherited matrix of gender relations” (Burchardt 2010:77) that reinforces inequality. Women may be motivated to adhere to these teachings as a way of maintaining a valued identity as a pious, humble, and respectable woman.

Dominant gender norms were also reinforced by the community and served as a form of social control. As Rhandzu (age 48, cohabiting) observed: “People don’t feel good seeing a man sweeping…seeing him helping me push a wheelbarrow.” The stigmatizing of a man helping a woman push a wheelbarrow underscores the gendered nature of domestic responsibilities such as farming and gathering water (both assigned to women) and highlights the community’s active role in sanctioning behaviors that challenge gendered expectations. Ellen (age 46, cohabiting) described men’s policing of each other regarding hierarchy in their relationships. When asked whether she and her partner practiced 50/50, Ellen replied:

I don’t use 50/50 with my husband^3^. He doesn’t like it…He said he won’t live it in his house, as he was telling his friends. I listened to him talk while telling his friends about their wives who are on 50/50. He said those men are giving their wives permission to be on 50/50 just because they [the men] are weak. It means their houses are ruled by their wives. I supported his statement as he was right.

There are few expectations for men’s involvement in household labor, and men who engage in tasks coded as feminine are regularly stigmatized and sanctioned (van den Berg et al. 2013; Dworkin et al. 2012). In describing men in 50/50 relationships as “weak” and “ruled by their wives”, Ellen’s husband upbraids his peers for not adhering to hegemonic masculinity norms and failing to uphold their power as men. This type of sanctioning can be an effective form of social control by bringing behavior back into alignment with dominant norms and serving as a cautionary tale (see also Horne and Mollborn 2020; Sennott and Angotti 2016). The combination of religious teachings on gender essentialism and community sanctioning reinforcing gendered expectations provided women a strong incentive to conform to dominant norms.

Another ideological explanation for women’s reaffirmation of gender inequality in relationships hinged on culture, respect, and tradition. Hlekani (age 23, single) described how 50/50 contradicts the local ideal of respectful womanhood and the fulfillment of tradition:

50/50 is proof that there is no more respect. A woman will tell her husband, “Hey, can you come with that cup?” Meanwhile in the past it was not like that. I have seen from my mother…She was cooking and after that she will bring the food to my father putting it on the smaller handmade table. She will kneel down [a sign of respect] and say, “You will eat the food, father.” But nowadays things have changed. They [women] don’t kneel and they just stand with food on the plate. That’s why there is a difference with our cultures. If a man can marry a Shangaan woman like us, we are well known for respect…Women told themselves that they are free. They don’t want to be under pressure…Like I said men need women who respect, women like Shangaans who are humble.

A woman’s dutifulness to her partner is an important tenet of acquiescent femininity in this context, and as illustrated by Hlekani’s comment that Shangaan women are “known for respect,” closely tied to women’s cultural identities. Further, by linking ideals of respectful feminine behavior to women “in the past,” Hlekani underscores the importance of fulfilling traditions to women attaining valued cultural identities, as opposed to women “nowadays” who are neither respectful nor cultural women. Thus, it was common for women to tie the “modern” practice of 50/50 to the erosion of culture, respect, and tradition.

Finally, ideological explanations for gender inequality in relationships were closely tied to the difficulty the local context presented for cultural change, as described by Glory (age 52, married):

In my community it’s rare to see people accepting 50/50…I think in our [rural] place like this one, it’s difficult to live the lifestyle of 50/50. 50/50 only applies to the location [urban areas] where most of the people are educated and they are working…I think the other thing that made us to be like this is the culture. Changing from the culture is still a challenge.

By distinguishing educated women in urban areas from rural women who embody cultural values and traditions, Glory distances herself and her community from 50/50. Like Hlekani, Glory claims a cultural and moral identity for rural women who embody dominant gender norms rather than pursuing 50/50. That is, women like Hlekani and Glory are staking a claim to a valued identity, and this act of prioritizing culture and tradition can be read as a form of agency (Mphaphuli and Smuts 2021). However, this agency cannot be understood apart from the significant constraints of the local context, as described below.

### Pragmatic Explanations for Gender Inequality

Pragmatic explanations for gender inequality in relationships focused on the strategies women employed to protect themselves from violence, HIV, and abandonment amidst local economic precarity. These explanations thus weighed women’s concerns about their health, wellbeing, and economic survival against concerns about gender imbalances in relationships. In doing so, women highlight the formidable structural constraints in the local political economy and health environment that serve as barriers to equality in relationships. This type of explanation is pragmatic in that women focused on the potential consequences of 50/50 rather than ideological motivations for dismissing 50/50.

Research demonstrates that challenges to men’s masculine identities and essentialized rights to power and control—such as through seeking gender equality in relationships—are often associated with efforts to shore up masculinity in other ways (Dworkin et al. 2012; Pierotti et al. 2018) including violence (Jewkes and Morrell 2012; Mphaphuli and Smuts 2021). Indeed, participants frequently linked 50/50 directly to the risk of IPV. For example, Gavaza (age 43, married) said violence was common in 50/50 relationships because “Most women [who use 50/50] want to be a boss to their husbands, and they end up fighting because men are the leaders of the household.” Similarly, Basani (age 20, single) said that a man may beat his wife “because she forces him to live by 50/50, meanwhile he doesn’t understand it.” These comments show the direct relationship women perceived between 50/50 and men’s violence. In another example, Fanisa (age 31, married) explained that 50/50 was related to violence because women in these relationships “don’t have a good approach. They start to undermine their husbands and think as if they are better than them.” Thus, in Fanisa’s view, women who used 50/50 were disrupting the normative gender order and therefore challenging men’s power. She went on to describe how this situation could result in violence or even death:

Fanisa: From there you will hear that they were fighting, and the woman has [moved] back to her parents’ home. Or you will hear that she was beaten to death by her husband.

Interviewer: Is that common nowadays?

Fanisa: This is very common…That’s where you will see that [violence] is not happening…in America only but even here locally things are happening. Women must be submissive to their husbands.

The threat of violence against women who support 50/50 in relationships serves as a form of social control that motivates women to abide by dominant gender norms. Further, this explanation elucidates the common view that 50/50 leads to violence because of women’s actions. Rather than blaming men for being violent, Gavaza, Basani, and Fanisa attribute men’s violence to a woman’s approach to implementing 50/50 by saying she “forces him” or “wants to be the boss.” This perspective aligns with a zero-sum approach to equality in relationships and leaves little ground from which women might negotiate for greater power.

Women also focused on how 50/50 could prompt men to have outside sexual affairs, which led to a heightened risk of HIV. For example, Alina (age 44, married) said: “…that [50/50] will lead a man to have an affair outside, where he will have peace of mind.” Alina’s comment about “peace of mind” highlights the belief that a man would leave a woman who was interested in 50/50 in favor of a woman who was more dutiful. Similarly, Priscilla (age 36, cohabiting) described how the push for 50/50 could encourage a man to seek a partner who would show him respect by attending to his needs:

If I’m abusing my man here at home by saying he needs to help me by doing the household chores…he will go out and find a charming girl and they will fall in love. From there he will pay a visit to that girl and he will find that there is no 50/50. She is giving him water to bathe and [he is] drinking tea while in bed and that girl is taking care of everything. Do you think he can come back to you, the woman of 50/50? I don’t think so. Men don’t want to be abused…He knows that with you he is working until late, and he doesn’t have time to rest, but with that girl he is able to rest.

As highlighted by Alina and Priscilla, men’s infidelity was characterized as an expected response to women’s attempts to have a more equal relationship. However, linking 50/50 to the “abuse” (Priscilla) of men, which was common across the sample, obfuscates the real risks of violence and HIV women face in seeking equality. Nearly half of individuals in Agincourt are HIV positive by the end of their reproductive years (Gómez-Olivé et al. 2013), therefore, remaining monogamous is an important way couples can avoid infection. This was reflected by Samara (age 38, married) who described a man’s reaction if a woman in a 50/50 relationship refuses sex: “If your husband wants to have sex with you and then you refuse, he will go out and start cheating, and it will hurt you and you will blame him. He will come back with infections [HIV], and he will infect you.” Comments like those from Alina, Priscilla, and Samara reinforce how the interrelated threats of infidelity and HIV incentivized women to adhere to norms dictating their submission. However, fulfilling the tenets of acquiescent femininity does not necessarily safeguard women from a partner’s infidelity or HIV transmission (see for example Jewkes and Morrell 2010; Sennott et al. 2020). Moreover, this approach ultimately relies on a gendered script that holds women responsible for how men treat them (see Gqola 2015; Mphaphuli and Smuts 2021).

Lastly, women offered a pragmatic explanation for reaffirming gender inequality in relationships by arguing 50/50 is only possible for women with a measure of economic independence. For example, Ripfumelo (age 35, married) said 50/50 was only good in couples where women were employed:

With us, as we are unemployed, it is not good to use it [50/50]. Even where the man is working and the woman is not, she should do everything in the household because she knows exactly what to do…Maybe I would use 50/50 if I was employed, but the fact is that at home I was taught about how I should behave when I am married so I won’t change myself.

Underscoring the significant challenges in the local political economy, both Ripfumelo and her husband were unemployed, which she viewed as a barrier to 50/50. This was common among the sample. Although Ripfumelo indicated she might seek more equality in her relationship if she was working, she ultimately falls back on her gendered responsibilities to her husband (“how I should behave when I am married”). In doing so, she prioritizes fulfilling norms of acquiescent femininity, something she can control, over pursuing a more equal partnership, an outcome she cannot fully control. Moreover, the former is a reliable path to respectability, regardless of whether she is working, whereas the latter may increase risk, as discussed above. Twelani (age 28, single) illustrates these dynamics in explaining how educated and uneducated women differ in terms of respect and 50/50:

Twelani: A woman who is not educated is full of respect, and if you want to see a man being happy just show him some respect, he will always be happy. And what I have realized is a woman who is not educated doesn’t live by 50/50 because she knows that she is not educated and not working. Once her husband can leave her, she will have nowhere to go, so that is why she will make sure that she respects her husband in all ways.

Interviewer: What about women who are educated?

Twelani: Mostly they don’t have respect to their men, do you know why? They have money. They are working, so there is nothing their man can say, because they have everything…Even when they can divorce, they will move on with their lives because they have money.

Twelani’s comments highlight the common perception that education and employment provide women with the space to negotiate for 50/50 as well as the economic resources and independence to survive if the relationship dissolves. These options are not necessarily open to women without the same resources, who instead may be compelled to rely on deference to men and the fulfillment of tradition to ensure economic stability and avoid abandonment (see also Mathis 2011; Sennott and Mojola 2017). Twelani and Ripfumelo also emphasize that women who use 50/50 lack respect. Thus, although education and employment may provide women with independence and the leverage to have more equal partnerships, doing so challenges norms and traditions surrounding respect and thus the identities of women *without* those resources. Ultimately, the pragmatic explanations for women’s reaffirmation of gender inequality in relationships underscore how the risks of violence, HIV, and abandonment encourage women to strategize about how to ensure their own security, safety, and stability.

Finally, of the 33 women who offered pragmatic explanations for reaffirming gender inequality, four did *not* ideologically support gender hierarchy and inequality in relationships^4^. Though their ideological views differed, their pragmatic explanations for reaffirming inequality echoed those above, namely the risks of abandonment (Maria, Olive), violence (Khongelani, Maria, Rhandzu), and infidelity and HIV (Khongelani). For instance, although Olive (age 41, single) felt that men and women “should be equal,” she said 50/50 was risky because:

Men don’t want 50/50. You cannot say “cook” or “take care of the children while I go somewhere.” Men don’t want that. They want to be free to walk and drink with friends. So, I don’t want to take chances…You will hear men who fought with their wives saying “It’s all about 50/50. She is doing that because she got powers. Just leave her like that [divorce her], you will see where she is going [back to her parents’ home].”

In saying she does not “want to take chances” by seeking a more equal relationship, Olive’s comments reflect the tension between ideological support for 50/50 and the risks of pursuing equality. Maria (47, cohabiting) offered a similar explanation for avoiding 50/50, even though she ideologically supported gender equality in relationships: “Men always want to be on top…If you tell him to come back home early, he will tell you that you can’t set his limits as he is the family head and it is his home…He will say, ‘This is my home. Go back where you are coming from.’“ Finally, despite her ideological support of 50/50 and her belief that men and women “need to share equally,” Khongelani (age 34, single) described why 50/50 was not practically feasible:

It’s the men. They are cheating…We are not feeling well about this but men don’t change…We are not equal as men are beating women for nothing. It’s like with me, the way [her previous boyfriend] treated me was not 50/50…Men are cheating and forgot that nowadays there is illness. I mean the illness of HIV. They are going to infect us.

For these women the risks of abandonment, infidelity, HIV, and violence, ultimately outweighed their ideological support for 50/50. These findings highlight the formidable barriers to embracing 50/50 given local conditions.

## Discussion and Conclusions

This study examined women’s views about gender equality in relationships in South Africa, a country in which democratic ideals and a governmental commitment to equality exist alongside extreme levels of inequality, gender norms that essentialize difference, a resurgence of tradition, and high rates of IPV and HIV (Ainslie and Kepe 2016; Mathis 2011; UNAIDS 2018; World Bank 2019). Given these contradictions, a growing body of research has examined the social and cultural factors upholding gender inequality, often focusing on norms about hegemonic masculinity and the backlash among men to women’s rights (van den Berg et al. 2013; Dworkin et al., 2012; Gibbs et al. 2015, 2020; Ratele 2015; Walker 2005; Willan et al. 2020). This has left a dearth of evidence about how women themselves—the targets of numerous governmental policies and programmatic efforts—view gender equality in relationships.

Our findings suggest that women in rural South Africa accounted for gender inequality in relationships on both ideological and pragmatic grounds. First, women based their ideological support of gender hierarchy and inequality on longstanding norms of gender essentialism that are widely supported by local institutions, community members, and women’s social networks. Thus, for many women, fulfilling the tenets of acquiescent femininity was closely tied to respect, culture, and tradition and thus their duty as women. These findings echo research from across sub-Saharan Africa showing that women in rural areas in particular are likely to defer to dominant norms due to their ideological commitments and the status these ideals provide, but also because of a lack of viable alternatives (Evans 2018; Pettifor et al. 2012; Wyrod 2008, 2016). For example, Evan’s (2018) research from Zambia showed that women in rural areas subscribed to dominant gender ideologies and restricted their aspirations to being wives and mothers largely because of limited exposure to alternative discourses and a lack of confidence in the likelihood of social change. Similarly, Campbell and Nair’s study in South Africa found that although women lamented the gender inequality they faced, they were unwilling to challenge men’s power because they viewed it as something to “work around” rather than something they could change (2014:1222). Our findings that women relied on gender essentialist scripts and the fulfillment of culture and tradition in their reaffirmation of inequality demonstrate that dominant norms about gender hierarchy in relationships can be particularly impervious to change.

However, our findings that women take a pragmatic approach to 50/50 to protect themselves from men’s violence, infidelity, HIV, and abandonment also show the limits of cultural explanations for the reproduction of gender inequality. That is, women in our study often weighed their concerns about relationship inequality vis-à-vis the uncertainties of living in a rural setting marked by structural and health disadvantages and a lack of opportunities. At times this was acknowledged directly in women’s assessments that 50/50 was only possible for those who were educated or working and thus had some measure of economic independence. In essence, women’s pragmatic explanations for avoiding 50/50 reflected the idea that rights cannot be realized without resources (Campbell and Nair 2014; Englund 2006).

Nonetheless, our findings emphasize that it is not just economic uncertainty that influences women’s views about equality in relationships, rather, it is the interrelationship between local norms supporting gender hierarchy, precarity in the political economy, and the substantial risks of violence and HIV. Extant research from South Africa underscores the challenge of reducing IPV where normative support for change is lacking. In work by Treves-Kagan and colleagues (2020), for example, culture and tradition were invoked as barriers to reducing violence (see also Dworkin et al. 2012, 2015; Hatcher et al. 2014). Thus, as we show, where economic uncertainty is rife, women may forgo gender equality in their relationships to avoid violence and a partner’s infidelity (Kandiyoti 1988). These findings emphasize the importance of embedding understandings of women’s beliefs about gender equality and their concomitant behaviors within local contexts by considering the political economic factors as well as health conditions—such as HIV/AIDS—that may complicate the adoption of equality in relationships (Kurzman et al. 2019; Thomas 2007; Unnithan and Pigg 2014).

Although our study captures how women in rural South Africa view gender equality, we are unable to discern the interpersonal processes through which women may gain more power in relationships. Research is necessary to explore, for example, whether women who forgo 50/50 because of pragmatic concerns would be more likely to implement it given different local conditions. Future research should also examine the strategies women employ to negotiate for more equal relationships and compare women across settings to determine how local contexts might differentially influence women’s views of equality and the extent to which they are able to challenge unequal gender relations. Because our study focuses on the views of women in rural South Africa, future research among women in peri-urban or urban areas would be particularly instructive in this regard. As Evans (2018) suggests—and supported by our participants—urban areas may offer more opportunities for patriarchal norms to be disrupted and therefore for women to challenge the structures that subordinate them. This type of analysis could also introduce more variation among participants in race, ethnicity, and social class, allowing for more axes of comparison. Our findings highlight the view that 50/50 may be more attainable for women with education or economic resources, suggesting that social class may be especially important to examine. Our research also reinforces the importance of continuing gender transformative work with men, which has already begun across many areas of South Africa (e.g., van den Berg et al. 2013; Dworkin et al. 2012, 2015). Recent research suggests that though progress has been slow, these interventions can help shift community norms and gender relations in a more equitable direction (Gibbs et al. 2015, 2020; Ratele 2015; Treves-Kagan et al. 2020; Willan et al. 2020). Including more women and networks of community members in these endeavors may enhance the likelihood of sustaining change (Treves-Kagan et al. 2020). Finally, future research on gender equality that includes extended kin would be insightful for understanding what other types of status and hierarchy—such as age or seniority—within family systems can influence women’s views and experiences of 50/50 in relationships and more broadly (Moore 2015; Oyewùmí 1997).

This study builds on and extends research from the Global North that has found that largescale change toward gender equality has been slowed by the persistence of longstanding gender norms (e.g., Ridgeway 2011). It is clear that broader structural changes, such as gender-progressive legislation, are necessary for more equitable societies; however, rights only go so far (Englund 2006; Hames 2006; Undie and Izugbara 2011). Despite the implementation of equal rights in South Africa, women in rural areas still face multiple structural barriers—alongside longstanding norms—that inhibit equality in relationships. We suspect that women across the Global South frequently contend with similar local material conditions and social structures that shape their views about equality and ultimately their health and wellbeing. In rural South Africa, for example, high rates of employment precarity and poverty, threats of gender-based violence, and the risk of HIV are omnipresent regardless of women’s ideological perspectives on gender equality. This suggests research focusing solely on cultural explanations for the persistence of gender inequality will be incomplete in overlooking pragmatic concerns. Therefore, our findings underscore the importance of incorporating both ideological and pragmatic factors in analyses of what inhibits or encourages greater equality in relationships in the Global South and beyond. We believe this approach has the potential to identify the complex interplay of local norms, material conditions, and risks to wellbeing that need to be addressed to effectively transform gender hierarchies and move closer to equality.

## Footnotes

1 These efforts are in response to the 2019 protests against the rising tide of violence against women in South Africa. See https://www.gov.za/blog/desk-president-36#.

2 Grace (age 23, single) described her views this way: “50/50 is good. I cannot do all the household activities meanwhile he [boyfriend] is sitting under the shadows. We must help each other. If I’m washing the dishes, he is sweeping the house, if I’m washing clothes, he is cooking.... We are helping each other. We are equal. It doesn’t mean…he doesn’t bathe [their son]. He has to.” Similarly, Gift (age 46, cohabiting) said: “I think it [50/50] helped us a lot as we were oppressed as women. Men were abusing us as we were not allowed to work, meanwhile they abandoned us. His parents also didn’t care about you as long as you woke up and did all the household activities. But now 50/50 allowed me to do [what I want] according to my will and without consulting him.”

3 Women often refer to their long-term or cohabiting partners as “husbands” even if they are not officially married.

4 All the women quoted above as offering pragmatic explanations also ideologically supported gender hierarchy in relationships.
